# Alternatives to Conventional Enterocystoplasty in Children: A Critical Review of Urodynamic Outcomes

**DOI:** 10.3389/fped.2013.00025

**Published:** 2013-10-07

**Authors:** Ricardo González, Barbara M. Ludwikowski

**Affiliations:** ^1^Department of Pediatric Surgery and Urology, Auf der Bult Kinder- und Jugendkrankenhaus, Hannover, Germany; ^2^Charité Universitätsmedizin Berlin, Virchow Klinikum, Berlin, Germany

**Keywords:** ureterocystoplasty, seromuscular colocystoplasty, autoaugmentation, enterocystoplasty, bioengineered bladder, detrusorectomy, detrusorotomy

## Abstract

Alternatives to conventional enterocystoplasty have been developed in order to avoid the most common complications derived from contact of the urine with intestinal mucosa. In this article critically we review the literature on the topics: ureterocystoplasty, detrusorectomy, detrusorotomy, seromuscular gastroenterocystoplasty, use of off the shelf biomaterials, and bladder augmentation by bioengineering. Recognizing the difficulty of deciding when a child with a history of posterior urethral valves requires and augmentation and that the development of a large megaureter in cases of neurogenic dysfunction represents a failure of initial treatment, we conclude that ureterocystoplasty can be useful in selected cases when a large dilated ureter is available. Seromuscular colocystoplasty lined with urothelium (SCLU) has been urodynamically effective in several series when the outlet resistance is high and no additional intravesical procedures are necessary. Seromuscular gastrocystoplasty lined with urothelium seems to offer no distinct advantages and involves a much more involved operation. The use of seromuscular segments without urothelial preservation, with or without the use of an intravesical balloon has been reported as successful in two centers but strict urodynamic evidence of its effectiveness is lacking. The published evidence argues strongly against the use of detrusorectomy or detrusorotomy alone because of the lack of significant urodynamic benefits. Two recent reports discourage the use of small intestinal submucosa patches because of a high failure rate. Finally, research into the development of a bioengineered bladder constructed with cell harvested from the same patient continues but is fraught with technical and conceptual problems. In conclusion of the methods reviewed, only ureterocystoplasty and SCLU have been proven urodynamically effective and reproducible.

Normal bladder capacity (BC) and compliance are essential to maintain normal renal function and allow urinary continence. The most frequent causes of reduced functional or anatomical BC in children are neurogenic dysfunction (NVD), posterior urethral valves (PUVs), and bladder exstrophy (BE). When more conservative methods (1) such as medications or injection of Botulinum toxin fail, surgical bladder augmentation is needed to achieve continence and prevent or correct hydronephrosis.

The use of reconfigured intestinal segments to increase bladder volume is effective (2, 3) but not without side effects (4, 5). Bringing urine in contact with functioning ileal or colonic mucosa can cause metabolic acidosis and intestinal mucus secretion into the urinary bladder may lead to difficult emptying and bladder lithiasis. Perforation of the augmented bladder can be a life threatening complication. The risk of malignancy development has been of concern to many but appears to be <5% (6).

Almost three decades ago, gastrocystoplasty was developed in hopes to obviate some of these problems (7, 8). Unfortunately the use of stomach had to be all but abandoned because of undesirable outcomes and side effects (3) in many patients (9).

Many researchers have explored alternatives to conventional enterocystoplasty. These include ureterocystoplasty (10), detrusorotomy and detrusorectomy (11), seromuscular enterocystoplasty with or without preservation of the urothelium (12, 13), the use of biomaterials (14) and most recently, attempts to construct a bioengineered bladder with cultured cells (15).

Although many very innovative and creative procedures have been reported some reports lack rigorous analysis of urodynamic data before and after the procedure. The degree of augmentation should be determined by comparing pre- and post-operative pressure specific BC or safe BC (16) and when appropriate, expected safe capacity for age. Here we report our analysis of the urodynamic outcomes of alternative procedures to conventional enterocystoplasty published in the literature in order to guide practicing surgeons as to the appropriate indications and expected results of the various available techniques.

## Materials and Methods

We reviewed the literature (PubMed) under the headings bladder augmentation, ureterocystoplasty, enterocystoplasty, autoaugmentation, non-secretory cystoplasty, and bioengineered bladder. Articles containing pertinent information were selected and when available, full text articles reviewed. Articles reporting post-operative BC are cited but only those reporting pre- and post-operative pressure specific BC (16) and age specific BC are discussed in detail. Series selected for this report comprised largely pediatric populations. Case reports are cited if they report on important technical innovation.

## Results

### Ureterocystoplasty

The use of a dilated ureter to enlarge the bladder was first described by Eckstein and Martin (10) but no reports followed until 1993 when the separate reports of Bellinger (17) and Churchill et al. (18) awakened widespread interest in this technique. The reasons for this interest are clear since the ureter is lined with urothelium avoiding the side effects related to urine being in contact with intestinal or gastric mucosa. All conceivable variations of ureterocystoplasty have been described including the use of the entire ureter and removal of the ipsilateral kidney (19), using only the distal ureter on one side and reimplanting the proximal ureter in the bladder (20), constructing a transureteroureterostomy (21), or using both distal ureters (22). The use of lower ureter of a completely duplicated in conjunction with a lower pole nephrectomy has been also reported (23). Peroviç et al. dilated the ureter distal to a loop ureterostomy prior to using it to augment the bladder (24).

Hitchcock et al. (21) reported urodynamic results in eight children (PUV4; BE2; NVD2) in seven of whom the ureterocystoplasty was done with a segment of one dilated distal ureter with preservation of the kidney and a transureteroureterostomy. BC increased from 100 ml (mean, range 45–215) to 311 ml (mean, range 150–450) a statistically significant difference. Pre- and post-operative bladder pressures decreased in all cases to safe values. This report was followed by a report from Miami (25) of five cases in which a variety of techniques were used resulting in a pressure specific BC increase from 142 ml (mean) to 500 ml.

Landau et al. (26) compared the results of ureterocystoplasty (eight children) and ileocystoplasty (eight children) and found a small difference in the degree of increased BC between the groups favoring ureterocystoplasty. In contrast, Podesta conducted a similar study and found ileocystoplasty to produce a greater degree of augmentation than ureterocystoplasty (27) but in both reports the results of uretero and ileocystoplasty were similar. A retrospective study of a larger series from two centers was reported in 1999 (28). Thirty-two patients had ureterocystoplasty either with the entire ureter of a one or two non-functioning kidneys or with a segment of dilated distal ureter the proximal end of which was then anastomosed to the contralateral ureter. The authors found no significant differences between the use of one or two ureters but reported a greater increase in pressure specific BC when the entire ureter was used (median increase in BC 3.75-fold) and a lesser increase when a segment of distal ureter was used (mean increase 230%). Unfortunately absolute BC in one group was not reported.

Husmann et al. (29) conducted a retrospective multicenter study including 64 patients operated with various techniques. The majority of the patients (71%) had NVD. They concluded that in patients with non-refluxing megaureters with an ultrasonographic diameter ≥1.5 cm had universal success when the entire ureter was used for the augmentation. In contrast, those with refluxing megaureters only did well if the compliance of the system was normal or only mildly decreased. In this series, the group of patients in whom a dilated distal ureter was used resulted in poor augmentation and the need to re-augment the bladder was 92%. This is in sharp contrast with the success rate reported in 22 patients using a segment of dilated distal ureter and by Pascual et al. (30). I n this series pressure specific BC increased from a mean of 105–254 ml (2.4-fold) with an improvement in the mean compliance from 10 to 24 ml/cm H_2_O. Half of the patients reached the expected capacity for age but one suffered a spontaneous perforation and underwent enterocystoplasty. The degree of augmentation is comparable to other series (see Table 1). Other reports already discussed had also obtained acceptable results using a segment of distal ureter (21, 28).

**Table 1:** **Summary of results of ureterocystoplasty in publications that listed pressure specific BC**.

Reference	Number of cases	Mean age (year)	PUV	NVD	BE	Other	Whole ureter	Distal ureter	Fold increase in pressure specific BC
Churchill et al. (18)	16	8.8	1	12	1	2	15	1	Unknown
	4 (With intra-operative ureteral occlusion)								2.84
Hitchcock et al. (21)	8	8.5	4	2	2		1	7	3
Gosalbez and Kim (25)	7	14	2	3				7	3.5
Zubieta et al. (28)	32	9	7	20		5	19	13	3.75^a^
									2.30^b^
Peroviç et al. (24)	16	6.6	8	8				8	3
Pascual et al. (30)	22	7.2		22				22	2.4
Husmann et al. (29)	64	Not stated	18	46			40	24	6 (Single non-refluxing ureter ≥1.5 cm)
									1.6 (Single refluxing ureter)
									All others failed
Podestá et al. (27)	8	6	1	5			8		1.83
Johal et al. (31)	17	5.9	10	2	3	2	7	9	2.3
Kajbafzadeh et al. (32)	13	7.3		7				7	2

^a^ Whole ureter.

^b^ Distal ureter.

FU, mean follow up period; PUVs, posterior urethral valves, NVD, neurogenic bladder dysfunction; BE, bladder exstrophy, BC bladder capacity.

In response to the report of Husmann at al. (29), Johal et al. (31) analyzed the results in 17 children followed for a mean of 4.5 years. The series was different from Husmann et al.’s in that only two children had NVD. Nine underwent augmentation with a distal dilated ureter and preservation of the kidney. Overall success was seen in 76% of the patients and four required enterocystoplasty.

Many others have documented an acceptable success rate with various forms of ureterocystoplasty (32–36).

Table 1 summarizes the results of 10 publications reporting pressure specific BC.

### Detrusorectomy/detrusorotomy

In 1953 Couvelaire proposed removing part of the detrusor leaving the urothelium intact as a means of increasing BC in tuberculous cystitis (11) and called the procedure bladder decortication. The concept of incising or removing part of the pathological detrusor to allow the more normal urothelium to expand and thus increase BC was described again by Cartwright and Snow (37). They called the procedure “bladder autoaugmentation.” The reported degree of augmentation confirmed in pre- and post-operative urodynamics studies after either detrusorectomy or detrusorotomy has been modest at best (38–44) (Table 2) and some reports showed a decrease in BC after the operation in almost 1/3 of the cases (45). In an experimental canine model of reduced BC, Garibay et al. reported no increase in BC 6 months after partial detrusorectomy (46).

**Table 2:** **Summary of results of detrusorectomy or detrusorotomy in publications that listed pressure specific BC**.

Reference	Number of cases	Mean age (year)	PUV	NVD	BE	Other	Fold increase in pressure specific BC	Comments
Stothers et al. (38)	12	4–14	1	10		1	0.4	Detrusorotomy
Carr et al. (44)	8						1.96	Peritoneal flaps
Oge et al. (39)	13	11.9		11		2	0.61	Detrusorectomy and peritoneal flap
MacNeily et al. (40)	17	10.2		17			1.4	71% Considered clinical failures
Lindley et al. (41)	11	10		11			1.3	Detrusorectomy covered with omentum or peritoneal flap
Rocha et al. (42). Group 1	8	12		7			0	Detrusorectomy
Rocha et al. (42). Group 2	10	8.5		10			1.7	Detrusorectomy and silicone balloon for 2 weeks
Veenboer et al. (43)	26	15		26			1.26	Detrusorectomy
Hansen et al. (47)	25	9		25			1.35	Pressure or age specific BC not reported
Total	130						Mean 1.05	

PUVs, posterior urethral valves; NVD, neurogenic bladder dysfunction; BE, bladder exstrophy; BC, bladder capacity.

Hansen et al. (47) reported long term results of detrusorectomy in 25 children with NVD followed for a mean of 6.8 years. BC was defined as “maximum tolerated bladder filling or volume at leakage.” Pressure specific BC was not reported. Mean maximal BC decreased in the first few months from 130 to 96 but increased progressively after 1 year to a mean of 176 ml in 19 of 25 patients. This represents an increase of 1.35-fold assuming that the mean BC for the 19 patients was the same as that of the entire group. Age specific expected BC was not reported. Compliance increased in the first 5 years of follow up in many patients. Of interest is that leak pressure decreased sharply in five children who had no additional bladder neck procedures. Table 2 summarized the series published.

Chrzan et al. (48) recently reported 49 patients with NVD in whom the preoperative expected BC for age was mean 98% (mean, range 35–153%). After detrusorectomy BC rose to 108% (15–156%). They authors claimed “good and fair” success in 39/49 cases (79.6%). Unfortunately raw urodynamic data were not reported making the interpretation of the data difficult. They do not explain why the operation was done in patients with normal or greater than normal BC. In the view of the authors the fact that 11 of 49 patients (22%) were able stop taking antimuscarinic medications seemed to justify the use of this procedure.

An interesting technical variation of detrusorectomy was reported by Rocha et al. (42). This group performed detrusorectomy in 22 children, in 12 in whom the standard technique was used, no urodynamic benefit was appreciated. In contrast, in the 10 children in whom an intravesical silicon balloon filled with 50–100 ml of fluid was left indwelling for 2 weeks, the expected BC for age increased from a mean of 46–82%. Regrettably, again, actual BCs for this group was children were omitted from the table published.

### Covered detrusorectomy

Disappointment with the results of detrusorectomy and detrusorotomy, which is likely due to the development of fibrosis around the denuded urothelium and regrowth of abnormal detrusor muscle (40, 46), led many to develop techniques to prevent fibrosis and preserve BC and compliance. Peritoneal flaps, omentum, rectus muscle, the seromuscular layer of the colon or the stomach have been used with varying success.

Although in the usual course of a detrusorectomy performed extraperitoneally the denuded urothelium ends up covered with the parietal peritoneum that normally covers the cephalad half of the bladder, Oge et al. (39) created a peritoneal flap to cover the urothelium and obtained only minimal improvement (Table 2). Carr et al. also covered the urothelium with peritoneal flaps with variable clinical results (44). Likewise, Lindley et al. obtained minimal augmentation effect covering the (41) denuded urothelium with omentum in 11 patients (Table 2). Peroviç et al. (49) covered the urothelium with rectus abdominis muscle flaps in seven children and reported a 2.27-fold increase in BC but unfortunately the definition of BC employed is not given.

### Seromuscular colocystoplasty lined with urothelium

In 1995 two independent groups from USA and Brazil published clinical series of an operation consisting of a detrusorectomy covered by a reconfigured seromuscular segment of left colon (12, 13). The US group called this procedure seromuscular colocystoplasty lined with urothelium (SCLU) and the Brazilian group used the term non-secretory sigmoid cystoplasty. The main difference among the techniques lies in the method to remove the colonic mucosa. Whereas the US technique preserved the colonic submucosa, the other removed it in an effort to prevent colonic secretory cells regrowth. Table 3 shows results of eight series published by four different groups of investigators who reported pre- and post-operative urodynamic data. The authors of these reports are from US, Europe, Turkey, and Korea. In all 147 patients have been reported with an average 2.64-fold increase in BC. González et al. (50) reported that success with this technique depends on being able to maintain the bladder distended for the first post-operative week to allow coaptation of the urothelium to the seromuscular sigmoid segment. They recommended keeping the pressure inside the bladder at about 20 cm H_2_O. This necessitates a water tight bladder mucosa. In this authors’ view, this fact limits the possibility of performing concomitant procedures that violate the urothelium such as appendicovesicostomy or ureteroneocystostomy. They reported universal success when there was no post-operative urine leak. In contrast, three of five patients who underwent a simultaneous appendicovesicostomy, had prolonged urinary leaks and failed. An experimental study indicated that the preserved urothelium in SCLU behaves normally regarding the absorption of ammonia (51).

**Table 3:** **Summary of results of seromuscular colocystoplasty line with urothelium in publications that listed pressure specific BC**.

Reference	Number of cases	Mean age (year)	NVD	PUV	Fold increase in BC	
Gonzalez et al. (12)	16	11.7	14	2	2.4	
Lima et al. (13)	10	10.4	9		2.49	Did not measure safe BC but maximal BC
Dewan (78)	6		6		2.9	
Dayanç et al. (76)	10	13	10		3.8	
Jednak et al. (66)	28	11.1	28		2.4	
González et al. (52)	27	11	27		2.36	All had AUS
González et al. (50)	16	9	16		1.83	Without concomitant bladder procedures
Jung et al. (77)	34	10			2.96	
Total	147				2.64	

PUVs, posterior urethral valves; NVD, neurogenic bladder dysfunction; BE, bladder exstrophy; BC, bladder capacity.

Also the procedure works best in patients with high outlet resistance to prevent early urine leak around the catheter, for example those who have an artificial urinary sphincter or a sling implanted prior or at the time of the augmentation (52). Preoperative measures of leak point pressure are no always reliable as the detrusorectomy can cause a drop in outlet resistance (38).

### Seromuscular gastrocystoplasty lined with urothelium

Instead of using the seromuscular layer of the colon, other authors have used the gastric wall from which the mucosa and submucosa had been removed in small clinical series. Unfortunately none of the published series report pre- and post-operative pressure specific BC. Dewan and Stefanek (53) reported on seven patients in whom the BC increased 5.9-fold. Nguyen at al. (54) reported seven patients with an increased in BC of 2.16-fold and Carr et al. (44) reported on 10 patients with urodynamic data in whom BC increased 1.85. There have been no reports on the clinical use of this operation in the last 14 years.

### Seromuscular enterocystoplasty without urothelial preservation

To avoid the sometimes tedious dissection necessary to separate the detrusor from the bladder urothelium when constructing a seromuscular augmentation lined with urothelium, Lima proposed opening the bladder in its full thickness as for a conventional enterocystoplasty and then augmenting it with a segment of colon (55) or ileum (56) from which the mucosa and submucosa had been removed. To prevent contraction of the intestinal patch, a silicon balloon was left in the bladder for 2 weeks. The first series reported using colon included 24 patients half of which were older than 10 years. The authors report an increase in BC of more than fourfold, however the data are difficult to interpret since the method to determine BC is not described and before the operation 21/24 had a capacity <100 ml of which three had 0 ml BC. These low values are seldom encountered in practice and in other publications. Another criticism of this publication is that the series included three children <4 years of age. The justification for doing an augmentation at such early age is not given. In the eight patients in whom the ileum was used, BC increased an average of 2.9-fold. Regrettably the method of measuring BC (i.e., pressure specific or maximal) was not specified in either of these reports. The same group published a summary of their experience with these techniques in 2008 (57). In a total of 173 patients with urodynamic data, 23 failed. In the remaining patients mean increase in capacity was threefold. The same shortcomings described for the 1998 paper are also present in this publication.

Like Lima, de Badiola et al. (58) also omitted preservation of the urothelium, did not find the use of a balloon necessary in 10 children and coagulated the sigmoid mucosa with the argon coagulator before removal to prevent regrowth. At a mean follow up of 18 months, they reported an increase in BC of 3.75-fold. The method used to determine BC is not reported. No other centers have reported results of these procedures.

### Patches of biomaterials

The idea of using a readily available off the shelf material has attracted investigators for decades. Only few clinical studies have been reported. Arikan et al. (14) reported the use of Dura matter in 10 patients with NVD to augment the bladder, which had been previously used experimentally by Kelâmi (59). They reported a 2.3-fold increase in BC but the follow up period was not mentioned. Caione et al. (60) used small intestinal submucosa (SIS) in five children after closure of BE and obtained a negligible increase in BC. Likewise Schaefer et al. (61) failed to obtain good results in five patients with a variety of diagnosis using SIS to en large the bladder. SIS had been used experimentally with encouraging results (62) which is just one example of the difficulty of moving from animal experimentation to the clinic.

### Bioengineered bladder

Experimental efforts to construct a tissue engineered bladder with a scaffold seeded with cultured cells previously obtained from the bladder to be augmented have been reported for 15 years (63, 64) and culminated with a report of its clinical application in seven patients with myelomeningocele (15). At the time of that report the mean follow up was 46 months. At 25–36 months of follow up BC decreased in one patient and there was a mild increase in three (1.04-, 1.24-, and 1.5-fold). At 49–61 months of follow up, four patients had urodynamic studies. Increase in BC was 1.56-, 1.02-, 1.53-, and 3.45-fold. Pressure specific BC was not reported and the compliance in this apparently successful case was only 10.2 ml/cm H_2_O and the bladder pressure 47 cm H_2_O. At last follow up the only patient with acceptable compliance (18 ml/cm H_2_O) was one who had a preoperative BC of 438 ml. No further clinical reports of this procedure have been published.

## Discussion

The objective results of any procedure used to augment BC should be measured urodynamically. Symptomatic results are difficult to interpret given the variable reporting practices regarding urinary continence. Neurologically impaired patients often have altered bladder sensation and detrusor sphincter dyssynergia or non-relaxing bladder outlet. In this population, measurement of BC based on patient’s desire to void or leak point (65) should be avoided. Instead, safe or pressure specific pressure should be used (16). Significant differences between maximal BC and safe BC have been reported (66). This should be taking into consideration when interpreting results of procedures since it often leads to an overoptimistic interpretation.

It should be kept in mind that the purpose of bladder augmentation is to provide a capacious, low pressure reservoir to maintain renal integrity and allow the patient to be dry emptying the bladder periodically by intermittent catheterization. Spontaneous voiding after bladder augmentation can be achieved in neurologically intact patients but it is the exception rather than the rule.

In this review we have included some series that reported total BC but have pointed out this potential shortcoming. In uncontrolled studies with long follow up in children it is difficult to differentiate the benefits from the procedure and the normal growth in BC.

### Ureterocystoplasty

From this review it is clear that patients needing a bladder augmentation who have a significantly dilated ureter can benefit from ureterocystoplasty. The operation can be done transperitoneally or retroperitoneally through one or two incisions or laparoscopic assisted (67). The papers reviewed present divergent opinions regarding the use of a segment of distal ureter versus the entire ureter. Of course, the latter is only possible when the kidney does not function and can be removed. One of the problems of most reports is that the preoperative urodynamic evaluation in patients with massive reflux can be difficult to interpret. Podesta et al. (27) used video-urodynamics in an attempt to attribute volumes and pressures to the bladder versus the ureters. Churchill et al. (18) and subsequently Gosalbez and Kim (25) occluded refluxing ureters intraoperatively to measure BC independently from the volume that refluxed into the ureters.

The finding by Husmann et al. (29) that in patients with refluxing ureters the results are good only when the compliance of the system (bladder plus refluxing ureters) is normal or mildly decreased raises the question whether the augmentation in those patients was really needed. Probably in such cases, only by occluding the ureters during the urodynamic investigation can the proper indication for augmentation be made. This method has the limitations of a study performed under general anesthesia. The question is particularly valid for most children with a history of PUV and massive uni or bilateral reflux. Furthermore, in PUV patients, BC and compliance tend to increase with age, particularly when there is associated polyuria. In general the indications for bladder augmentation are not clearly defined. A bladder that may have inadequate capacity in a polyuric child with renal failure may be perfectly adequate after renal transplantation when the daily urine output normalizes (68).

The situation is quite different in patients with NVD. In this patient population, good care from the onset should prevent hydronephrosis and the development of massive ureteral dilatation. Some authors have reported acceptable results in such patients but is should be kept in mind that the development of a useful megaureter in children with NVD should be an exceptional occurrence (32).

In order to expand the application of ureterocystoplasty, researchers have progressively dilated normal ureters in experimental animals hoping to make them useful for bladder augmentation but clinical application’s have not been reported (69, 70).

In conclusion, ureterocystoplasty can be useful in selected patients; however, in the most common condition in childhood needing bladder augmentation, namely NVD, it should rarely be an option. In the case of children with a history of PUV, a condition frequently associated with megaureters, the indications for bladder augmentation are often unclear (68).

### Detrusorectomy/detrusorotomy

Although the term auto augmentation has become widely accepted it should be abandoned. In Greek *autós* means self or spontaneous and augmentation implies increase or enlargement, therefore the meaning of either term does not apply to the operations more correctly termed detrusorotomy or partial detrusorectomy. Although a few groups around the world continue to perform these operations, the degree of urodynamic augmentation reported by most is minimal or non-existing raising serious doubts about the justification of its use (40, 41, 47, 71, 72). In our opinion, the fact that in one report a handful of children stopped taking anticholinergic medications (48) does not justify the operation. The idea of keeping the bladder distended after the detrusorectomy with a balloon as has been reported for other forms of cystoplasty (see below) is intriguing and hopefully it will be further explored by Rocha et al. (42) and reported with more valid urodynamic data. From the literature reviewed it is apparent that covering the urothelium with peritoneal flaps or omentum does not improve outcomes significantly. The small series from Belgrade (24) in which the urothelium was covered with rectus muscle flaps is interesting but has not been reproduced by others and a larger series with longer follow up has not been reported.

### Seromuscular enterocystoplasty

The concept of a detrusorectomy covered with a segment of intestine without mucosa to create an *in vivo* bioengineered bladder seems to have been developed simultaneously in the US (12) and Brazil (13). Dewan and Stefanek in Australia used a segment of the stomach without mucosa for the same purpose in four patients (53). The same authors published a case report of seromuscular augmentation colocystoplasty preserving the urothelium in a single patient (73). It is interesting that this patient was operated on and reported despite unfavorable results of this procedure in a sheep model obtained by the same authors on the same year (74). In contrast, both the US and the Brazilian authors performed the procedure in patients after successful experimentation in canine models (13, 75). The results of SCLU, the operation described by González et al. (12) have been reproduced by surgeons in Turkey (76), Korea (77), and Australia (78). Bandi et al., independently of the surgeon who performed the procedure, compared 26 cases of SCLU with 32 of conventional enterocystoplasty and found a comparable degree of bladder augmentation. In all 147 cases have been reported and the degree increase in safe BC was 2.6-fold. One of the limitations of this operation is that it requires to maintain the bladder distended for 1 week after surgery to allow coaptation of the urothelium to the seromuscular colonic segment. This implies having high outlet resistance by prior or simultaneous implantation of an artificial sphincter or a sling in patients with NVD since high leak point pressures may change after detrusorectomy (38, 47). It is also desirable to avoid post-operative urine leaks (50). The use of a gastric segment to cover the denuded epithelium seems to offer no advantages over the colon and has the potential for increased morbidity. Thus it cannot be recommended.

There is a difference of opinion as to whether the submucosa of the colon should be left in place. Both Lima et al. (79) and Dewan et al. (80) recommended removing it to prevent regrowth of colonic epithelium, but Buson el al. (75) argued that removing the submucosa leaves an unsatisfactory bowel segment for augmentation.

To avoid the spending the time and effort necessary to perform a satisfactory detrusorectomy leaving the urothelium intact, Lima et al. (79) and de Badiola et al. (58) used the demucosalized bowel as in a conventional cystoplasty, that is without preserving the urothelium. To prevent contraction of the segment, Lima et al. left an intravesical silicone balloon indwelling for 2 weeks. The reported results of their series did not include changes in safe BC and the procedure has not been reported from other centers. Likewise, the removal of the intestinal mucosa with the argon coagulator has not been reported by other groups (58).

### Patches of biomaterials

Despite promising initial results with the use of a lyophilized patch of Dura matter by one author (14), no other reports have followed. Clinical results with the use of SIS suggest that this operation should not be done (60, 61).

### Bioengineered bladder

The creation of a suitable graft composed of a scaffold lined with cultured urothelial cell inside and detrusor myocytes outside than can then be implanted in a patient to augment the bladder is a feat of tissue engineering which has potential clinical applications. It would be unquestionably of benefit to have large amounts of cultured urothelium from a given patient to apply as a lining to a flap of tissue capable of improving BC and compliance. Such approach, using cultured urothelium and a seromuscular colonic segment has been used experimentally (81). However, a construct of detrusor myocytes grafted into a patient with abnormal bladder innervation is doomed to fail since if it were re-innervated it would end up with the same problems as the original bladder. Furthermore inducing re-vascularization and re-innervation of such constructs has been problematic. An excellent review on the subject has recently been published (82).

## Conclusion

This review suggests that the only alternatives to enterocystoplasty for which there is some evidence of urodynamic effectiveness are ureterocystoplasty and SCLU. Both techniques require good patient selection and are applicable to only some of the patients requiring enterocystoplasty. It is to be hoped that further research in this field will produce other alternative in the future.

## Conflict of Interest Statement

The authors declare that the research was conducted in the absence of any commercial or financial relationships that could be construed as a potential conflict of interest.

## Abbreviations

BC, bladder capacity; BE, Bladder exstrophy; NVD, neurovesical dysfunction; PUVs, posterior urethral valves, SCLU, seromuscular colocystoplasty lined with urothelium.
